# Novel chemical library screen identifies naturally occurring plant products that specifically disrupt glioblastoma-endothelial cell interactions

**DOI:** 10.18632/oncotarget.4957

**Published:** 2015-07-22

**Authors:** Rajarshi Sengupta, Amy Barone, Jayne Marasa, Sara Taylor, Erin Jackson, Nicole M. Warrington, Shyam Rao, Albert H. Kim, Jeffrey R. Leonard, David Piwnica-Worms, Joshua B. Rubin

**Affiliations:** ^1^ Department of Pediatrics, Washington University School of Medicine, St. Louis, Missouri, USA; ^2^ High Throughput Screening Core, Washington University School of Medicine, St. Louis, Missouri, USA; ^3^ Department of Neurosurgery, Washington University School of Medicine, St. Louis, Missouri, USA; ^4^ Molecular Imaging Center, Mallinckrodt Institute of Radiology, Washington University School of Medicine, St. Louis, Missouri, USA; ^5^ Department of Anatomy and Neurobiology, Washington University School of Medicine, St. Louis, Missouri, USA; ^6^ Department of Cell Biology and Physiology, Washington University School of Medicine, St. Louis, Missouri, USA; ^7^ Department of Cancer Systems Imaging, University of Texas MD Anderson Cancer Center, Houston, Texas, USA

**Keywords:** perivascular niche, GBM, high throughput screen, co-culture, iridin

## Abstract

Tumor growth is not solely a consequence of autonomous tumor cell properties. Rather, tumor cells act upon and are acted upon by their microenvironment. It is tumor tissue biology that ultimately determines tumor growth. Thus, we developed a compound library screen for agents that could block essential tumor-promoting effects of the glioblastoma (GBM) perivascular stem cell niche (PVN). We modeled the PVN with three-dimensional primary cultures of human brain microvascular endothelial cells in Matrigel. We previously demonstrated stimulated growth of GBM cells in this PVN model and used this to assay PVN function. We screened the Microsource Spectrum Collection library for drugs that specifically blocked PVN function, without any direct effect on GBM cells themselves. Three candidate PVN-disrupting agents, Iridin, Tigogenin and Triacetylresveratrol (TAR), were identified and evaluated in secondary *in vitro* screens against a panel of primary GBM isolates as well as in two different *in vivo* intracranial models. Iridin and TAR significantly inhibited intracranial tumor growth and prolonged survival in these mouse models. Together these data identify Iridin and TAR as drugs with novel GBM tissue disrupting effects and validate the importance of preclinical screens designed to address tumor tissue function rather than the mechanisms of autonomous tumor cell growth.

## INTRODUCTION

Despite decades of clinical and basic research, a diagnosis of glioblastoma (GBM) continues to carry a dismal prognosis, and new approaches to cure are needed. Recent studies have identified a sub-population of tumor cells with enhanced tumor-initiating capability, known as cancer “stem-like” cells (CSCs). CSCs are thought to drive tumor growth and recurrence [1-5], and therefore CSC-directed therapy may provide a long-awaited critical advance in GBM care. CSCs are localized to a specialized domain that surrounds the tumor microvasculature, often referred to as the peri-vascular niche (PVN). The PVN is a complex structure composed of tumor cells, microglia, astrocytes, pericytes, and endothelial cells [6, 7]. Each component cell type may play a role in the maintenance of the CSC phenotype, thereby promoting tumor growth and therapeutic resistance [8-11]. Therefore, successful ablation of CSCs may be possible by targeting their interactions with these non-tumor cell components of the PVN.

To better define the mechanisms by which endothelial cells drive GBM growth, and to provide a system for high throughput screening for drugs that can disrupt the functions of the PVN, we developed a co-culture system in which we could measure the tumor-promoting effects of endothelial cells on GBM cells [8]. In this model, primary cultures of human brain microvascular endothelial cells (HBMECs) and either an established human GBM cell line (U87) or primary GBM cell isolates were cultured together in a laminin-rich extracellular matrix (Matrigel). In Matrigel, HBMECs adopted a phenotype and spatial distribution reminiscent of endothelial cells *in vivo*. Addition of either primary GBM or U87 cells to the HBMECs resulted in migration of tumor cells to the PVN where they exhibited enhanced growth. A trophic effect of the PVN was mediated by endothelial cell-derived CXCL12 [8] and was blocked by treatment with the CXCR4 antagonist AMD3100, depletion of CXCL12 in endothelial cells or overexpression of G-protein coupled receptor kinase 3, a negative regulator of the CXCL12 receptor, CXCR4, in tumor cells [12]. These studies support the functionality of this model and its application in efforts to both identify pathways that mediate endothelial and GBM cell cross-talk, and compounds that can target PVN function. We hypothesized that screens incorporating elements of this critical cell-cell interaction would have a higher likelihood of identifying agents with significant *in vivo* activity.

A cell based high-throughput drug screen offers the potential to identify novel compounds that can be quickly moved to pre-clinical evaluation. Furthermore, examination of the targets of these lead compounds may reveal previously unappreciated biologic pathways contributing to GBM growth. We used our co-culture system to screen the Spectrum Collection compound library (Microsource Discovery Systems). This library contains a bio-diverse group of 2000 compounds including FDA approved drugs, compounds that are currently in clinical trials, experimental agents and natural extracts. Recent high-throughput screens of this library have identified potential novel anti-glioma therapeutics [13, 14]. However, our screen is distinct from these prior studies as it measures anti-tumor cell effects in the setting of tumor-endothelial cell co-culture. Since endothelial cells can induce a treatment resistant and pro-growth state in tumor cells [15], we hypothesized that drugs that affect tumor cell growth in this more “native” microenvironment would have a greater chance of blocking tumor growth *in vivo*. Specifically, our current study was designed to identify drugs that inhibit glioma growth by disrupting the interaction between glioma and endothelial cells within the PVN rather than acting on tumor cells alone.

## RESULTS

Our previous studies demonstrated that an established GBM cell line, as well as tumor cells derived from primary pediatric GBM, showed enhanced growth when co-cultured with HBMECs in a laminin-rich extracellular matrix [8]. This suggested that incorporation of these tumor microenvironmental elements would support a significantly more clinically relevant assessment of novel candidate anti-GBM agents. We used our co-culture system to perform a high throughput compound library screen to identify novel compounds that could specifically block the trophic effects of endothelial cells on GBM cells. We designed the screen to rule out compounds that are simply cytotoxic to GBM cells and to identify those that are both non-toxic to tumor cells directly and potent antagonists of the trophic effect of the endothelia on tumor cell proliferation.

### Primary screens

**Figure 1:**
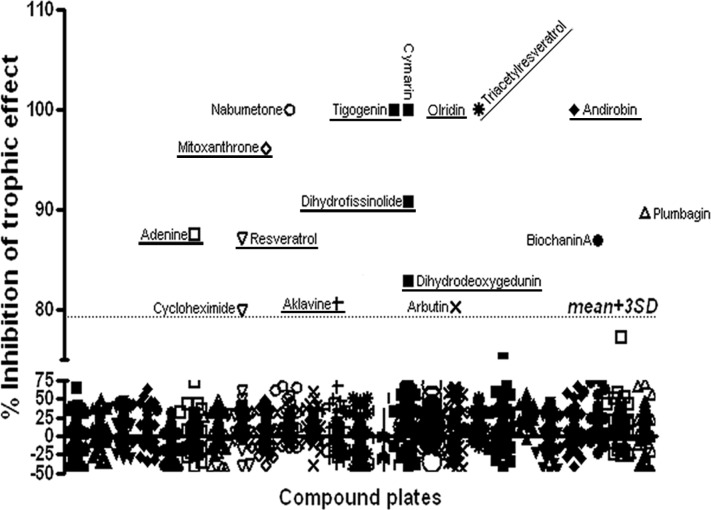
Compound Library Screen Results: Two thousand compounds in the Spectrum Collection were screened for their efficacy in blocking the trophic effect of co-culture on luciferase-expressing U87 cell growth (% inhibition of trophic effect) Dotted line indicates three standard deviations above the mean effect. Compounds with inhibitory effects greater than 3 SD above the mean are identified. Those compounds with both inhibitory effects greater than 3 SD above the mean and no direct cytotoxic effect are underlined.

The initial screens were performed using a glioblastoma cell line (U87) stably expressing a GFP-Luciferase construct in which U87 cell number has been demonstrated to be linearly related to bioluminescence (BLI) [16, 17]. Similar to our previous reports [8, 12], when cultured with HBMECs, U87 cells exhibited a ~2 fold increase in tumor cell number as measured by BLI. When delivered at the compound library standard dose of 5μM, drug effects on HBMEC-stimulated U87 growth fell into four categories. The majority of compounds had no effect on U87 cells alone and did not block the trophic effect of endothelial cells on U87 cells (Figure 1, Supplemental Figure 1). A second small group of compounds were generally cytotoxic to U87 cells alone and in co-culture. These were ruled out for further evaluation, as their anti-tumor effect was not specific to disrupting PVN function. A third interesting group was cytotoxic to U87 cells in monoculture, but not when U87 cells were co-cultured. Based on the protective effects of the endothelial cells, this group of drugs, listed in Supplemental Table 1, would not likely exhibit *in vivo* anti-tumor activity, and these results highlight a pitfall of monoculture drug screening. The final class of drugs was a small but diverse group of compounds that had no effect on U87 monocultures but significantly blocked the trophic effects of HBMECs on U87 cells. Compounds with an anti-trophic effect of greater than three times the standard deviation of the mean library effect and without any direct cytotoxic effect were prioritized for additional evaluation (Table 1). Ten compounds met these criteria. Among them were two anthracycline anti-neoplastic agents, aklavine and mitoxanthrone. Interestingly, mitoxanthrone has recently been demonstrated to have efficacy in recurrent GBM [18, 19]. Also included were Dihydrodeoxygedunin, a member of a compound family with known neural differentiating activity [20] and both resveratrol and its derivative, Triacetylresveratrol. Resveratrol has garnered much attention as a potential anti-aging and anti-neoplastic agent [21-23].

**Table 1:** Candidate PVN disrupting agents

Compound				% Inhibition*	Reported targets	
TIGOGENIN				100	p38 MAPK	
IRIDIN				100	Unknown activity	
TRIACETYLRESVERATROL				100	P53, Notch	
ANDIROBIN				100	Unknown activity	
MITOXANTHRONE HYDROCHLORIDE				96	Antineoplastic, Topo II inhibitor	
DIHYDROFISSINOLIDE				90	Unknown activity	
ADENINE				87	Vitamin B4	
RESVERATROL				87	Activates Notch-1, block Src/STAT3	
DIHYDRODEOXYGEDUNIN				83	Unknown activity	
AKLAVINE HYDROCHLORIDE				80	antibacterial, antineoplastic	

* The % inhibition of trophic effect by each compound was calculated as follows: %inhibition = [BLI (vehicle treated co-culture) − BLI (drug-treated co-culture)]/BLI (vehicle treated co-culture) − BLI (monoculture)] × 100

### Secondary screens

Only four compounds, Tigogenin, Iridin, Triacetylresveratrol (TAR) and Andirobin completely blocked the trophic effects of endothelial cells without any direct cytotoxic effects on the U87 cells. Therefore, these compounds were evaluated in secondary screens in which we sought first to first identify *in vitro* activity against a panel of primary adult and pediatric GBM specimens. These secondary screens were designed to directly test the dose responses to each compound in cell systems with greater fidelity to native GBM cell biology and with which we could capture the heterogeneity of GBM as it occurs in children and adults. We first determined whether the compounds might have toxicity against normal human astrocytes as this could limit their development as clinical agents. We treated primary human astrocyte cultures with each drug (5 μM) and found that similar to their effects on U87 cells these compounds were non-toxic in monoculture (Supplemental Figure 2). As primary GBM cells did not contain luciferase, we could neither measure GBM cell number using BLI nor readily distinguish changes in GBM and endothelial cell number in physical co-culture. We therefore developed an alternate approach for assays of endothelial cell effect on primary GBM cell number involving primary GBM cell culture in media conditioned by HBMECs. In pilot studies, primary pediatric GBM cells (CDI-2, 3 and 4) were cultured in either standard tumorsphere media (TSM) or tumorsphere media conditioned by HBMECs for 96 hours. Cell numbers were measured using a cell proliferation kit (Promega). HBMEC conditioned media (CM) induced tumor cell growth to a similar extent (~2-5 fold) as physical co-cultures with U87 cells and primary GBM cells as we previously reported (Supplemental Figure 3A) [8]. To determine whether there was any specificity in this growth effect for neoplastic cells, we measured growth of normal human astrocytes and found that HBMEC conditioned media had only a small effect on their growth (1.2 fold) suggesting some specificity of the HBMEC effect on GBM cells (Supplemental Figure 3B). These data indicated that factors secreted by HBMECs more potently stimulate GBM compared to astrocyte cell growth. Therefore, we performed initial secondary screens by treating tumor cells grown in CM in the presence or absence of the anti-trophic compounds. As a negative control we used melatonin, a compound that was completely inactive in the primary screen.

Similar to their activity in the primary screen, none of the drugs had any direct effect on the tumor cells themselves but exhibited significant potency in blocking the trophic effect of the CM. The anti-trophic effects of the drugs were cell-type specific. Tigogenin, Iridin and TAR successfully blocked the trophic effect of CM on CDI-2 cells and had a partial effect on CDI-4 cells, but were completely ineffective with the CDI-3 cells (Supplemental Figure 3A). Andirobin was ineffective at blocking the trophic effects of CM on any of these primary GBM specimens (data not shown). These results suggest that Andirobin may block a contact-mediated, rather than a secreted factor-mediated, effect of ECs on GBM cell growth. We recently reported induction of phosphodiesterase 7B in GBM tumor cells through direct contact-mediated effects of endothelial cells in this same co-culture model [24]. Andirobin was not further evaluated in these studies. Together, these results emphasize the importance of multiple cell line testing to address the highly heterogeneous nature of GBM. In line with our observations from the initial screens, melatonin had no effect on primary GBM cells in the secondary screen.

**Figure 2:**
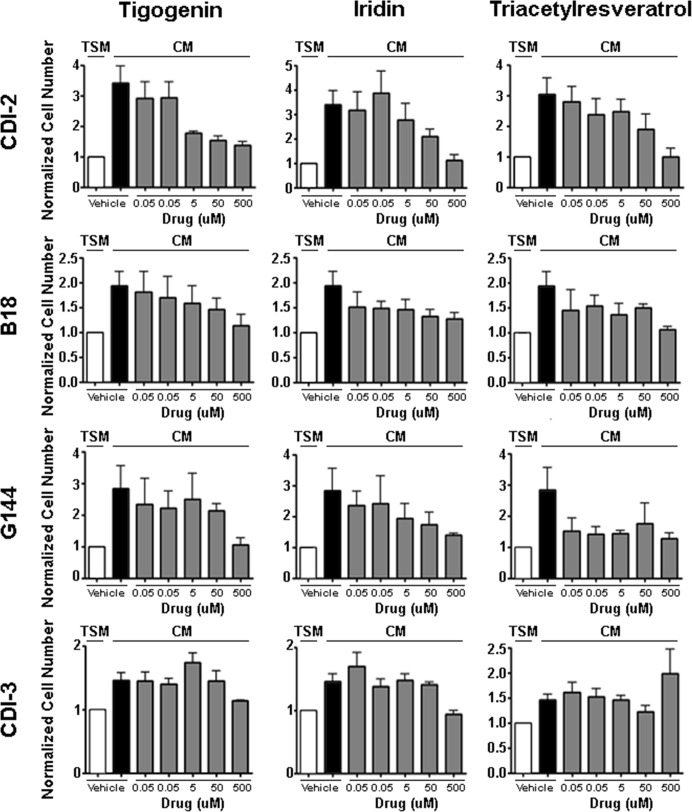
Dose Response Curves for Lead Compounds in Primary GBM Cultures: Tigogenin, Iridin and TAR were each tested against a panel of primary GBM cultures (CDI-2, B18, G144, CDI-3) In each case, drug efficacy was measured by its ability to block the trophic effect of HBMEC conditioned media (CM). The basal trophic effect was measured as the fold-increase in cell number induced by CM (compare white to black bars). Cell number measured in CM cultures treated with a range of drug doses as indicated were normalized to cell number in equivalent drug treated TSM cultures. Shown are the means and SEM of three independent experiments.

To determine the optimal doses for the anti-trophic effect of these compounds, we performed dose response studies for Tigogenin, Iridin and TAR on the pediatric GBM cell lines CDI-2 and CDI-3, as well as two additional primary adult GBM derived stem cell lines, G144 and B18 (Figure 2). We tested the anti-trophic activity of these drugs at doses ranging from 0.05μM to 500 μM. Tigogenin and Iridin displayed reproducible dose responses in the CDI-2, G144 and B18 cells with maximal inhibition at 500 μM. Triacetylresveratrol had a similar dose response in the CDI-2 cells but exerted a general inhibition at most concentrations in G144 and B18 cells. Consistent with the pilot studies, none of the drugs showed any anti-trophic effect on CDI-3 cell proliferation. In primary human astrocytes, only Triacetylresveratrol demonstrated inhibition of the modest tropic effect of CM (Supplemental Figure 3C). These data confirmed the potential importance of these three compounds as candidate anti-GBM agents and also indicated that they exert their effects directly on tumor cells, but only when these tumor cells are sensitized through the actions of endothelial cell-derived secreted factors.

### Effect of compounds on HBMECs

In the primary physical co-culture screens, both tumor cells and endothelial cells were exposed to the anti-trophic compounds, but only the proliferation of tumor cells was assessed. To directly assess the effect of compounds on HBMECs, HBMECs were cultured with drugs at concentrations of 0.05-50 μM for 4 days (Supplemental Figure 4). Iridin and Tigogenin increased HBMEC proliferation by 30-40% at concentrations at or above 5 μM for Iridin and 50 μM for Tigogenin. In the primary screen (drug concentrations of 5 uM), Iridin and Tigogenin diminished the trophic effect of HBMECs on GBM cells, despite the possible increase in HBMEC cell number. At the dose used in primary screens (5 uM), HBMEC cell number was diminished by TAR by 35%. Therefore, the direct toxicity of TAR on HBMECs may account for some of the anti-trophic effect of that drug in the co-culture model of the primary screen. However, in the secondary screens, the experimental paradigm does not subject HBMECs to anti-trophic compounds, as the compounds are added to media conditioned by untreated HBMECs. Therefore, while the direct toxicity of TAR on HBMECs may account for some of its anti-trophic effect on tumor cell growth in the primary screen, the secondary screen demonstrates that TAR is also effective in diminishing the HBMEC trophic effect caused by direct action on tumor cells.

### Pre-clinical evaluation of lead compounds

**Figure 3:**
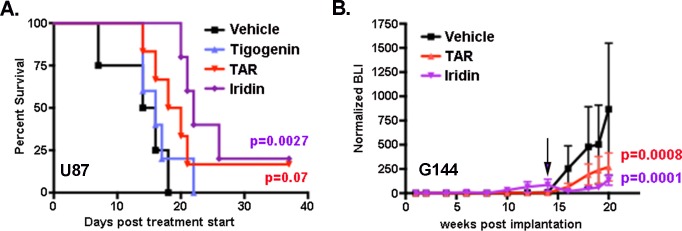
Iridin and TAR have significant *in vivo* anti-tumor effects **A.** Median survival in mice bearing intracranial xenografts of U87 cells was significantly prolonged by Iridin treatment. Median survival trended towards prolongation in Triacetyresveratrol (TAR) treated mice but was unaffected by Tigogenin treatment. P values were determined by Log-Rank test of Kaplan-Meier plot. **B.** Intracranial growth of luciferase-expressing G144 xenografts was followed by bioluminescence imaging (BLI) every other week. Shown are the mean +/− SEM BLI as a function of weeks post tumor implantation for three treatment groups (Vehicle, TAR-treated and Iridin-treated). Means were calculated by first normalizing each BLI measurement to the first BLI measurement for each mouse individually and then averaging the normalized BLIs for each treatment group. Initiation of treatment at week 14 is indicated by the arrow. Significance was determined by two-way ANOVA.

The ultimate goal of these studies is the identification of PVN-disrupting drugs for clinical evaluation. Thus, the anti-tumor effects of Tigogenin, Iridin and TAR were further evaluated using intracranial xenograft models of GBM as previously described by us and others [8, 25]. An initial *in vivo* screen was performed with U87 cells. As is standard in our model, 50,000 firefly luciferase-expressing tumor cells were stereotactically implanted into the cortex of nude mice. Engraftment and initial growth were verified by weekly bioluminescence imaging (BLI), and those mice with tumors exhibiting equivalent rates of growth were randomized into four treatment groups: 1) vehicle control, 2) Tigogenin, 3) Iridin and 4) TAR. Each drug was delivered by oral gavage at 20 mg/kg daily for 5 days each week. Compared to vehicle treated controls, Iridin treatment resulted in a statistically significant prolongation of median survival from 15 to 22 days (*p* = 0.0027, log-rank test) (Figure 3A). TAR treatment trended towards a similar survival benefit with a shift in median survival from 15 to 19 days (*p* = 0.07, log-rank test) compared to control. In this model, Tigogenin was without effect.

To further evaluate the potential therapeutic value of Iridin and TAR, we treated mice bearing intracranial xenografts of the primary G144 GBM isolate engineered to express firefly luciferase. After establishing engraftment and steady tumor growth (14 weeks post implantation), mice were randomized to three treatment groups: 1) vehicle, 2) Iridin and 3) TAR. Treatment was the same as for the U87 experiment, and intracranial growth was followed with bi-weekly bioluminescence imaging. Both Iridin and TAR rapidly and significantly blocked further intracranial growth (Figure 3B).

## DISCUSSION

Despite decades of research, median survival for GBM remains relatively unchanged. Novel therapeutics and approaches are needed. While reports of new drugs with promising activity are frequent, few gains have been observed in the clinic. Among the reasons for the disappointing performance of standard preclinical studies may be the common format for drug screening in which novel compounds are tested against panels of tumor cell monocultures to identify agents that can block tumor cell autonomous mechanisms of growth. GBM, like other solid cancers, are highly complex tissues in which cell-cell interactions drive tumor growth and promote resistance [6]. Thus, we reasoned that a screen for compounds that blocked these cell-cell interactions would enhance the specificity for drugs that could function *in vivo*, within the context of tumor tissue, and thereby improve the translational potential of any results.

To prove the utility of this approach, we purposefully focused on agents that had no direct cytotoxic effect and whose *in vitro* activity was confined to blockade of endothelial cell stimulated growth. We screened the Spectrum Collection because among the 2000 compounds in this library are many naturally occurring and FDA approved drugs and therefore there is potential for rapid translation. Several of the compounds were generally toxic and killed tumor cells grown alone or as co-cultures, some were toxic in monocultures but had no effect in the presence of endothelial cells, and only a few were capable of specifically blocking the trophic effect (> 3 SD of the mean) of endothelial cells on the GBM cells. These observations, particularly those instances where drugs were cytotoxic in tumor cell monocultures but not in the co-cultures, highlight the importance of the co-culture and HBMEC-conditioned media models for this screen. Treating tumor cells alone would have identified drugs with less translational potential.

Iridin, Tigogenin and TAR are all non-cytotoxic compounds derived from naturally occurring plant sources. Iridin is a glucoside found in both the rhizomes of the iris plant such as *Iris versicolor,* and the roots of violets. It is more commonly known as Blue Flag and has historically been used for “removing bile” and as a laxative. Multiple compounds derived from the Iridaceae family have been evaluated for their anti-tumor and anti-inflammatory effects [26]. Liu *et al.* showed that several have an anti-tumor effects on various cancer cell lines including stomach cancer, breast cancer, and prostate cancer [27]. Interestingly, Iridin did not exhibit a substantial anti-tumor effect in these monoculture assays, again underscoring the importance of drug screening in a co-culture system. It was only in this setting that Iridin's anti-tumor effect was revealed.

TAR is a derivative of Resveratrol with improved bioavailability [28]. Resveratrol has gained media attention for its potential anti-aging and anti-cancer effect. It was first discovered in 1937 in the roots of Japanese Knotweed, but it can also be derived from grape skins leading some to investigate its role in the health benefits seen from drinking red wine [29, 30]. Although anti-aging effects have not been consistently reproducible, it has been shown to have anti-inflammatory and blood sugar lowering effects [29, 30]. It has been evaluated in clinical trials for type 2 diabetes, Alzheimer's disease, cardiovascular disease, obesity, inflammation, concussions, and polycystic ovarian syndrome. Several studies have also indicated that Resveratrol may inhibit events associated with tumor formation and progression [31, 32]. Mgbonyebi *et al.* reported that Resveratrol inhibited the proliferation of human breast epithelial cells in a dose-dependent manner. Jang *et al.* showed that Resveratrol had anti-initiation, anti-promotion and anti-progression activity against human promyleocytic leukemia cells. Piceatonnol, a natural metabolite of Resveratrol, has also been shown to enhance cisplatin-induced apotosis, making it an attractive modulator for treatment of many cancers, especially ovarian cancer, which is often resistant to cisplatin [33]. Interestingly, Resveratrol has also been shown to decrease cell growth through inhibition of Notch 1 signaling [34], a pathway that has been shown to be important in growth of GBM and proliferation of GBM stem-like cells within the PVN [35]. These studies have led to clinical trial investigations in several malignancies such as gastrointestinal tumors, colon cancer, and multiple myeloma [36]. Resveratrol is readily available, making it an attractive therapeutic for a clinical trial in patients with GBM.

These studies suggest that the mechanisms of tumor cell expansion within tumor tissue can be distinct from the mechanisms that drive tumor cell expansion in monoculture. Thus, TAR and Iridin blocked U87 and primary GBM isolate growth in co-culture and *in vivo* but were without effect in monoculture. Rigorous elucidation of their mechanisms of action will be key to the continued development of these agents. This will include both the definition of their cellular targets and their intracellular target pathways. We tested the effect of each compound on both normal human astrocytes and HBMECs and found that only TAR exhibited any cytotoxicity and this effect was limited to HBMECs. In addition TAR was able to block the modest trophic effect of CM on astrocytes. Together these data suggest that the drugs are working primarily on GBM cells. Further elucidation of their target pathways is likely to advance our understanding of PVN biology and identify new therapeutic targets.

Finally, these results suggest that contextualized GBM cell growth carries specific vulnerabilities for tumor cells. It may be necessary to screen in this “tumor tissue” context to fully capitalize on the vast numbers of synthesized and available compounds before we can achieve significant improvements in the treatment of GBM and other intractable cancers. The data reported in this study directly supports this conclusion. Moreover, our data indicate that both Iridin and TAR are viable candidates for novel clinical trials designed to disrupt the function of the PVN. Particularly important may be the combination of PVN disruption with cytotoxic or targeted therapeutics.

## MATERIALS AND METHODS

### Ethics statements

#### Animal studies

All animals were used in accordance with an Animal Studies Protocol (# 20120174) approved by the Animal Studies Committee of the Washington University School of Medicine per the recommendations of the Guide for the Care and Use of Laboratory Animals (National Institutes of Health).

#### Human studies

Primary human GBM specimens for culture were obtained and utilized in accordance with a Washington University Institutional Review Board (IRB)-approved Human Studies Protocol (#201102299).

### Cell culture

#### Primary human GBM (CDI) cells

Fresh brain tumor resection material from pediatric glioblastoma patients was obtained according to a Washington University School of Medicine IRB approved human studies protocol. Resection material was minced into small pieces using sterile scalpels and dissociated in Accutase at 37°C. Single cells were obtained and cultured in tumor sphere media (TSM), which contains Neurobasal-A media (Gibco) supplemented with Glutamax (Gibco), 20 ng/mL epithelial growth factor (EGF, Sigma), 20 ng/mL basic fibroblastic growth factor (bFGF, Chemicon), 20 ng/mL leukemia inhibitory factor (LIF, Chemicon), 1 x N2 Supplement (Gibco), 1 x B-27 Serum-Free Supplement (Gibco), and heparin (20 ug/mL, Sigma). Cells were initially plated on tissue-culture coated plates overnight to allow non-stem-like cells to attach, and the non-adherent stem-like cells were transferred to extracellular matrix protein (ECM)-coated tissue culture plates prepared by coating with 10% ECM (Sigma) in Hanks Buffered Saline Solution (HBSS) and washed three times in HBSS. Thereafter, GBM stem cells were maintained in adherent culture on ECM-coated plates in TSM media, which was changed every 2 to 3 days.

#### B18 and G144 cells

Primary GBM cell lines were created from freshly isolated tumor resection tissue as previously described [37]. Briefly, primary GBM tumor tissue was cleaned manually of RBCs, mechanically dissociated with forceps and scalpel, and chemically dissociated with Accutase (Sigma). Cells were then spun down and triturated gently. Cells were then plated on PLO (Sigma) and Laminin (Sigma) coated Primaria plates (BD Biosciences). Cells were used for experiments after the fifth passage. Media is RHB-A with EGF (10ng/mL) and FGF (10ng/mL).

#### Primary human endothelial cells

Primary human brain microvascular endothelial cells (HBMEC) were obtained from ScienCell, Carlsbad, CA. HBMECs were used between passages 3-8 and maintained in endothelial cell growth media (EGM-2MV (Lonza, Basel, Switzerland)) on gelatin-coated dishes.

#### Primary human astrocytes

Primary normal human astrocytes (NHAs) were obtained from Lonza. NHAs were used between passages 3-8 and maintained in astrocyte growth media (EGM BulletKit (Lonza)) on Primaria plates.

#### Established human GBM cell line

U87 cells were originally obtained from ATCC and were engineered at low passage (< 5) to express a fusion protein of firefly luciferase and enhanced green fluorescent protein (eGFP) driven by the human ubiquitin C promoter after transduction with a lentivirus (FUW-FLG) as described previously [38-40]. U87 cells expressing firefly luciferase-eGFP (U87-Luc) were sorted to purity based on GFP expression, expanded and stored at −150 degrees Celsius. All experiments were performed with U87-Luc cells at less than passage 15 approximately 4 months post acquisition from ATCC. U87 cells were maintained in DMEM supplemented with 10% fetal bovine serum.

### GBM - endothelial cell co-cultures and high throughput screening

Human brain microvascular endothelial cells were plated (3000 cells/well) within an extracellular matrix (Matrigel, BD Dickinson) in 96-well plates compatible with bioluminescent measurement. After 24 hours (to allow for endothelial cell tubule formation), U87 cells (3000 cells/well) were added to the assay plates in minimal essential media as previously described [8]. Compounds from the Spectrum Collection (Microsource Discovery Inc.) were added to the co-cultures as well as to monocultures of tumor cells. DMSO vehicle treated wells at the same concentration as the diluted solvent in the library wells served as controls for each plate tested. The library consisted of FDA approved drugs (50%), natural products (30%) other non-drug bio-active compounds (20%). The compounds were supplied as 10mM solutions in DMSO and were used at a final concentration of 5μM for the assay. Each compound was tested in triplicate. Hence for each compound plate, we had six experimental (three tumor monoculture and three co-culture) plates. The High Throughput Screening Core at the Washington University School of Medicine was used for the screen. Cell numbers were assessed by BLI after 2 days in co-culture. The numbers of metabolically active tumor cells were determined by measuring luminescence upon addition of the substrate luciferin to the 96 well plates. The trophic effect was determined as the ratio of the mean of the bioluminescence reading from the co-cultures to that of the monocultures for vehicle treated wells. The % inhibition of trophic effect by each compound was calculated as follows: %inhibition = [BLI (vehicle treated co-culture) − BLI (drug-treated co-culture)]/BLI (vehicle treated co-culture) − BLI (monoculture)] × 100.

### Preparation of HBMEC conditioned media

#### Secondary screens

Primary GBM specimens or NHAs were cultured in ECM (Sigma) coated 96 well plates in either TSM or HBMEC conditioned media in the absence or presence of compounds. HBMEC conditioned media was generated daily by incubating an equivalent number of HBMECs with TSM overnight. After harvest of conditioned media, Iridin, Tigogenin, or TAR were added at concentrations from 0.05 μM to 500 μM. Media was changed daily for 4 days. After 4 days, cell proliferation was determined by the CellTiter 96 Aq_ueous_ One Cell proliferation Assay System following the manufacturer's protocol (Promega, Madison, WI). Absorbance at 490 nm was measured using the μQuant microplate Reader (Bio-Tek instruments, Winooski, VT). Absorbances from culture medium and CellTiter 96 Aq_ueous_ One Solution reagent served as background.

### Generation of intracranial xenografts

Intracranial xenografts were generated as previously described [16, 17, 40]. Tumor cell lines were harvested in mid-logarithmic growth phase and resuspended in PBS. Homozygous NCR female nude mice (Taconic Farms, Germantown, NY) were anesthetized with ketamine hydrochloride at 150 mg/kg and xylazine at 12 mg/kg (Phoenix Pharmaceuticals, St Joseph, MO) via intraperitoneal (i.p.) injection. The cranium was exposed, and a small hole was made with a size 34 inverted cone burr (Roboz, Githersburg, MD). Mice were fixed in a stereotactic frame (Stoelting, Wood Dal, IL), and cells were injected through a 27-gauge needle over 2 minutes at 2mm lateral and posterior to the bregma and 3mm below the dura (50,000 U87 cells in 7.5uL of PBS or 94,200 G144 primary cells in 6 uL of media). The incision was closed with Vetbond (3M, St. Paul, MN).

### Bioluminescence imaging

Bioluminescence imaging of intracranial xenografts was performed as previously described [16, 40]. NCR nude mice bearing intracranial xenografts of U87-luc cells were injected with 150 ug/g D-luciferin (Biosynth) in PBS, anesthetized with 2.5% isoflurance and imaged with a charge-coupled device camera-based bioluminescence imaging system (IVIS 50; Perin-Elmer, Waltham, MA; exposure time = 1-60 s, binning = 8, field of view = 12, f/stop = 1, open filter). Signals were displayed as photons/s/cm2/sr. Regions of interest were defined manually at 95% of the maximum pixel output using Living Image an IgorPro Software (v 2.50) and data were expressed as total photon flux (photons per second). Generally, the first mouse images were obtained 3-5 days following intracranial inoculation of the tumor cells then weekly. Data were analyzed and plotted as the ratio of bioluminescence on a given treatment day over bioluminescence on the first day.

### *In vivo* drug treatment

Mice bearing U87 intracranial xenografts were imaged twice after implantation to identify those with equivalent tumor growth rates. Two weeks after tumor cell implantation, cohorts of mice with approximately equivalent tumor bioluminescence were divided into equal control and treatment groups (5-6 mice per group). Mice bearing primary G144 xenografts were imaged every other week for three months to establish equivalent and continuous rates of growth. *Systemic therapies*: Tigogenin, Iridin, TAR (Microsource Discovery Systems), or vehicle was administered daily Monday through Friday (20mg/kg) by gavage. Drugs were resuspended in 0.5% carboxymethyl cellulose (CMC).

### Statistical analysis

Data were analyzed using GraphPad Prism version 4.00 (GraphPad Software). Kaplan-Meier survival curves were analyzed using pairwise log-rank tests. Given the repeated measurement of mice over time, statistical differences in growth curves were analyzed using the generalized estimating equation regression analysis.

## SUPPLEMENTARY MATERIAL FIGURES AND TABLE



## References

[1] Dean M, Fojo T, Bates S (2005). Tumour stem cells and drug resistance. Nature reviews Cancer.

[2] Kusumbe AP, Bapat SA (2009). Cancer stem cells and aneuploid populations within developing tumors are the major determinants of tumor dormancy. Cancer research.

[3] Mannino M, Chalmers AJ (2011). Radioresistance of glioma stem cells: intrinsic characteristic or property of the ‘microenvironment-stem cell unit’?. Molecular oncology.

[4] Nakai E, Park K, Yawata T, Chihara T, Kumazawa A, Nakabayashi H, Shimizu K (2009). Enhanced MDR1 expression and chemoresistance of cancer stem cells derived from glioblastoma. Cancer investigation.

[5] Quesnel B (2008). Tumor dormancy and immunoescape. APMIS : acta pathologica, microbiologica, et immunologica Scandinavica.

[6] Brooks MD, Sengupta R, Snyder SC, Rubin JB (2013). Hitting Them Where They Live: Targeting the Glioblastoma Perivascular Stem Cell Niche. Current pathobiology reports.

[7] Filatova A, Acker T, Garvalov BK (2013). The cancer stem cell niche(s): the crosstalk between glioma stem cells and their microenvironment. Biochimica et biophysica acta.

[8] Rao S, Sengupta R, Choe EJ, Woerner BM, Jackson E, Sun T, Leonard J, Piwnica-Worms D, Rubin JB (2012). CXCL12 mediates trophic interactions between endothelial and tumor cells in glioblastoma. PloS one.

[9] Galan-Moya EM, Le Guelte A, Lima Fernandes E, Thirant C, Dwyer J, Bidere N, Couraud PO, Scott MG, Junier MP, Chneiweiss H, Gavard J (2011). Secreted factors from brain endothelial cells maintain glioblastoma stem-like cell expansion through the mTOR pathway. EMBO Rep.

[10] Liu H, Patel MR, Prescher JA, Patsialou A, Qian D, Lin J, Wen S, Chang YF, Bachmann MH, Shimono Y, Dalerba P, Adorno M, Lobo N, Bueno J, Dirbas FM, Goswami S (2010). Cancer stem cells from human breast tumors are involved in spontaneous metastases in orthotopic mouse models. Proc Natl Acad Sci U S A.

[11] Zhu TS, Costello MA, Talsma CE, Flack CG, Crowley JG, Hamm LL, He X, Hervey-Jumper SL, Heth JA, Muraszko KM, DiMeco F, Vescovi AL, Fan X (2011). Endothelial cells create a stem cell niche in glioblastoma by providing NOTCH ligands that nurture self-renewal of cancer stem-like cells. Cancer research.

[12] Woerner BM, Luo J, Brown KR, Jackson E, Dahiya SM, Mischel P, Benovic JL, Piwnica-Worms D, Rubin JB (2012). Suppression of G-protein-coupled receptor kinase 3 expression is a feature of classical GBM that is required for maximal growth. Mol Cancer Res.

[13] Hothi P, Martins TJ, Chen L, Deleyrolle L, Yoon JG, Reynolds B, Foltz G (2012). High-throughput chemical screens identify disulfiram as an inhibitor of human glioblastoma stem cells. Oncotarget.

[14] Badr CE, Wurdinger T, Tannous BA (2011). Functional drug screening assay reveals potential glioma therapeutics. Assay and drug development technologies.

[15] Borovski T, Beke P, van Tellingen O, Rodermond HM, Verhoeff JJ, Lascano V, Daalhuisen JB, Medema JP, Sprick MR (2013). Therapy-resistant tumor microvascular endothelial cells contribute to treatment failure in glioblastoma multiforme. Oncogene.

[16] Goldhoff P, Warrington NM, Limbrick DD, Hope A, Woerner BM, Jackson E, Perry A, Piwnica-Worms D, Rubin JB (2008). Targeted inhibition of cyclic AMP phosphodiesterase-4 promotes brain tumor regression. Clinical cancer research : an official journal of the American Association for Cancer Research.

[17] Rubin JB, Kung AL, Klein RS, Chan JA, Sun Y, Schmidt K, Kieran MW, Luster AD, Segal RA (2003). A small-molecule antagonist of CXCR4 inhibits intracranial growth of primary brain tumors. Proc Natl Acad Sci U S A.

[18] Boiardi A, Silvani A, Eoli M, Lamperti E, Salmaggi A, Gaviani P, Fiumani A, Botturi A, Falcone C, Solari A, Filippini G, Di Meco F, Broggi G (2008). Treatment of recurrent glioblastoma: can local delivery of mitoxantrone improve survival?. Journal of neuro-oncology.

[19] Nava F, Tramacere I, Fittipaldo A, Bruzzone MG, Dimeco F, Fariselli L, Finocchiaro G, Pollo B, Salmaggi A, Silvani A, Farinotti M, Filippini G (2014). Survival effect of first- and second-line treatments for patients with primary glioblastoma: a cohort study from a prospective registry, 1997-2010. Neuro-oncology.

[20] Jang SW, Liu X, Chan CB, France SA, Sayeed I, Tang W, Lin X, Xiao G, Andero R, Chang Q, Ressler KJ, Ye K (2010). Deoxygedunin, a natural product with potent neurotrophic activity in mice. PloS one.

[21] Gagliano N, Aldini G, Colombo G, Rossi R, Colombo R, Gioia M, Milzani A, Dalle-Donne I (2010). The potential of resveratrol against human gliomas. Anti-cancer drugs.

[22] Borriello A, Bencivenga D, Caldarelli I, Tramontano A, Borgia A, Zappia V, Della Ragione F (2014). Resveratrol: from basic studies to bedside. Cancer treatment and research.

[23] Tresguerres IF, Tamimi F, Eimar H, Barralet J, Torres J, Blanco L, Fernandez-Tresguerres JA (2014). Resveratrol as anti-aging therapy for age-related bone loss. Rejuvenation research.

[24] Brooks MD, Jackson E, Warrington NM, Luo J, Forys JT, Taylor S, Mao DD, Leonard JR, Kim AH, Piwnica-Worms D, Mitra RD, Rubin JB (2014). PDE7B is a novel, prognostically significant mediator of glioblastoma growth whose expression is regulated by endothelial cells. PloS one.

[25] Sadahiro H, Yoshikawa K, Ideguchi M, Kajiwara K, Ishii A, Ikeda E, Owada Y, Yasumoto Y, Suzuki M (2014). Pathological features of highly invasive glioma stem cells in a mouse xenograft model. Brain tumor pathology.

[26] Ibrahim SR, Mohamed GA, Al-Musayeib NM (2012). New constituents from the rhizomes of Egyptian Iris germanica L. Molecules.

[27] Liu M, Yang S, Jin L, Hu D, Wu Z (2012). Chemical constituents of the ethyl acetate extract of Belamcanda chinensis (L.) DC roots and their antitumor activities. Molecules.

[28] Torres P, Poveda A, Jimenez-Barbero J, Ballesteros A, Plou FJ (2010). Regioselective lipase-catalyzed synthesis of 3-o-acyl derivatives of resveratrol and study of their antioxidant properties. Journal of agricultural and food chemistry.

[29] Baur JA, Pearson KJ, Price NL, Jamieson HA, Lerin C, Kalra A, Prabhu VV, Allard JS, Lopez-Lluch G, Lewis K, Pistell PJ, Poosala S, Becker KG, Boss O, Gwinn D, Wang M (2006). Resveratrol improves health and survival of mice on a high-calorie diet. Nature.

[30] Lagouge M, Argmann C, Gerhart-Hines Z, Meziane H, Lerin C, Daussin F, Messadeq N, Milne J, Lambert P, Elliott P, Geny B, Laakso M, Puigserver P, Auwerx J (2006). Resveratrol improves mitochondrial function and protects against metabolic disease by activating SIRT1 and PGC-1alpha. Cell.

[31] Jang M, Cai L, Udeani GO, Slowing KV, Thomas CF, Beecher CW, Fong HH, Farnsworth NR, Kinghorn AD, Mehta RG, Moon RC, Pezzuto JM (1997). Cancer chemopreventive activity of resveratrol, a natural product derived from grapes. Science.

[32] Mgbonyebi OP, Russo J, Russo IH (1998). Antiproliferative effect of synthetic resveratrol on human breast epithelial cells. International journal of oncology.

[33] Farrand L, Byun S, Kim JY, Im-Aram A, Lee J, Lim S, Lee KW, Suh JY, Lee HJ, Tsang BK (2013). Piceatannol enhances cisplatin sensitivity in ovarian cancer via modulation of p53, X-linked inhibitor of apoptosis protein (XIAP), and mitochondrial fission. The Journal of biological chemistry.

[34] Zhang J, Chen J, Xu C, Yang J, Guo Q, Hu Q, Jiang H (2014). Resveratrol inhibits phenotypic switching of neointimal vascular smooth muscle cells after balloon injury through blockade of Notch pathway. Journal of cardiovascular pharmacology.

[35] Zhu TS, Costello MA, Talsma CE, Flack CG, Crowley JG, Hamm LL, He X, Hervey-Jumper SL, Heth JA, Muraszko KM, DiMeco F, Vescovi AL, Fan X (2011). Endothelial cells create a stem cell niche in glioblastoma by providing NOTCH ligands that nurture self-renewal of cancer stem-like cells. Cancer research.

[36] Carter LG, D'Orazio JA, Pearson KJ (2014). Resveratrol and cancer: focus on *in vivo* evidence. Endocrine-related cancer.

[37] Pollard SM, Yoshikawa K, Clarke ID, Danovi D, Stricker S, Russell R, Bayani J, Head R, Lee M, Bernstein M, Squire JA, Smith A, Dirks P (2009). Glioma stem cell lines expanded in adherent culture have tumor-specific phenotypes and are suitable for chemical and genetic screens. Cell stem cell.

[38] Warrington NM, Gianino SM, Jackson E, Goldhoff P, Garbow JR, Piwnica-Worms D, Gutmann DH, Rubin JB (2010). Cyclic AMP supppression is sufficient to induce gliomagenesis in a mouse model of Neurofibromatosis-1. Cancer research.

[39] Smith MC, Luker KE, Garbow JR, Prior JL, Jackson E, Piwnica-Worms D, Luker GD (2004). CXCR4 regulates growth of both primary and metastatic breast cancer. Cancer research.

[40] Yang L, Jackson E, Woerner BM, Perry A, Piwnica-Worms D, Rubin JB (2007). Blocking CXCR4-Mediated Cyclic AMP Suppression Inhibits Brain Tumor Growth *In vivo*. Cancer research.

