# Supplementary material for: Novel chemical library screen identifies naturally occurring plant products that specifically disrupt glioblastoma-endothelial cell interactions
Source: Oncotarget. 2015 Jul 22;6(21):18282–92. doi: 10.18632/oncotarget.4957 (PMC4621891; doi:10.18632/oncotarget.4957)
Supplement: Supplementary file 1 [file oncotarget-06-18282-s001.pdf]

Novel chemical library screen identifies naturally occurring plant products that specifically disrupt glioblastoma-endothelial cell interactions

Supplementary Material

Sengupta, Barone et al. Supplemental Figure 1

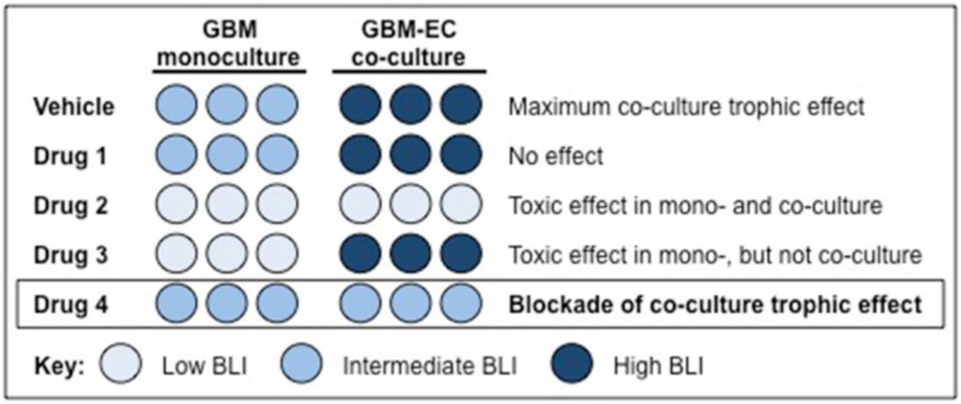

Supplemental Figure 1: Categories of Compound Library Screen Results. Each compound in the Spectrum Collection was tested in triplicate in U87 monoculture (GBM monoculture) and U87-endothelial cell co-culture (GBM-EC co-culture). Bioluminescence (BLI) results were normalized to vehicle controls. Maximum co-culture trophic effects were observed with vehicle treatment. Four categories of results were observed as illustrated by Drugs 1-4. 1) Drugs without effect did not alter BLI of mono- or co-cultures. 2) Drugs that were generally toxic decreased mono- and co-culture BLI. 3) Some drugs were toxic in monocultures but not in co-cultures. These drugs decreased monoculture but not co-culture BLI. 4) Drugs that specifically blocked the co-culture trophic effects had no effect on monoculture BLI and reduced co-culture BLI.

Sengupta, Barone et al Supplemental Figure 2

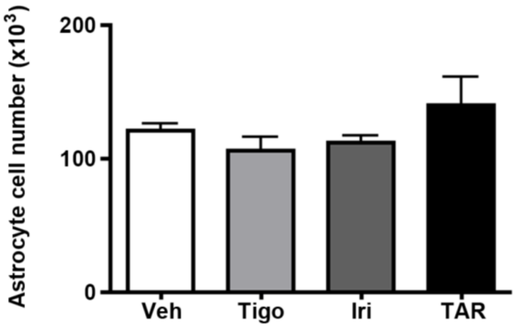

Supplemental Figure 2: Lead compounds are not toxic to normal human astrocytes. Primary cultures of human astrocytes (100,000 cells) were plated in astrocyte media and treated with vehicle (veh), Tigogenin (Tigo), Iridin (Iri) or Triacetylresveratrol (TAR). Cell number was measured at 48 hours. Shown are the means and SEM of three independent experiments.

Sengupta, Barone et al Supplemental Figure 3

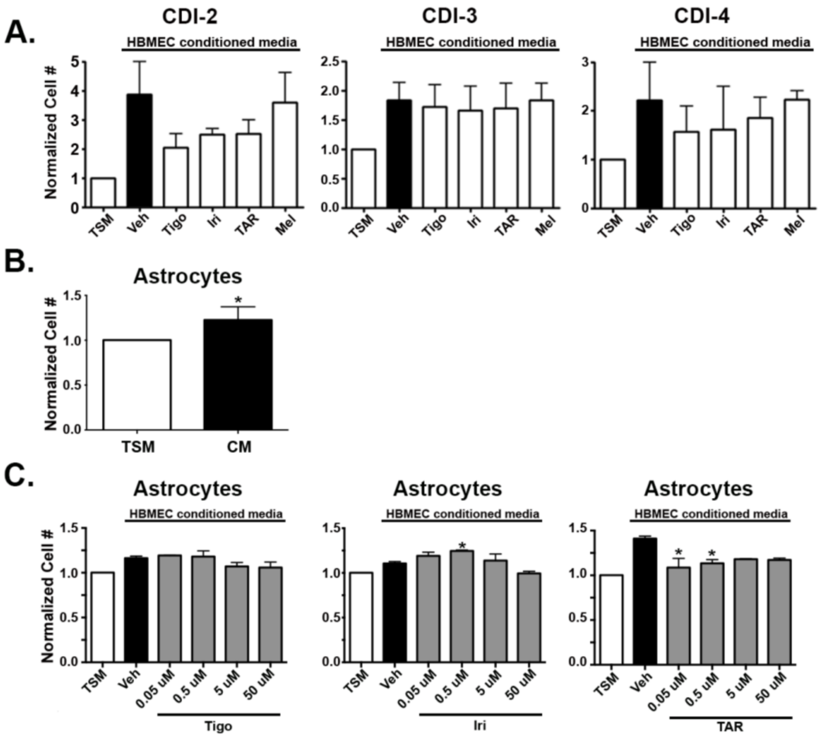

Supplemental Figure 3: Endothelial cell conditioned media contains trophic factors for GBM cells. (A) Three low passage primary pediatric GBM cell lines (CDI-2, CDI-3, CDI-4) were treated with tumorsphere media (TSM) or media conditioned by HBMECs for 96 hours alone (Veh) or HBMEC conditioned media containing 5 μM Tigogenin (Tigo), Iridin (Iri), Triacetylresveratrol (TAR) or Melatonin (Mel). Trophic effects were measured by cell counting normalized to control cell numbers (TSM). Data are presented as the means and SEM of three independent experiments with each cell line. Tigogenin, iridin and TAR exhibited growth inhibitory effects on CDI-2 and CDI-4 responses to CM but not on CDI-3 growth responses. Melatonin was without effect in any of the lines. (**B**) Primary human astrocytes exhibit a slight trophic response when treated with HBMEC-conditioned media (CM) vs. TSM alone (TSM). * indicates $p<0.05$ vs. TSM control using unpaired t-test. (**C**) Iridin, Tigogenin, and TAR were tested for their ability to inhibit the trophic effect of HBMEC-conditioned media on human astrocytes. The basal trophic effect was measured as the fold-increase in cell number induced by CM (compare white to black bars). Cell number measured in CM cultures treated with a range of drug doses as indicated were normalized to cell number in equivalent drug treated TSM cultures. Shown are the means and SEM. * indicates $p<0.05$ vs. vehicle using one-way ANOVA analysis with Tukey's multiple comparisons test.

Sengupta, Barone et al Supplemental Figure 4

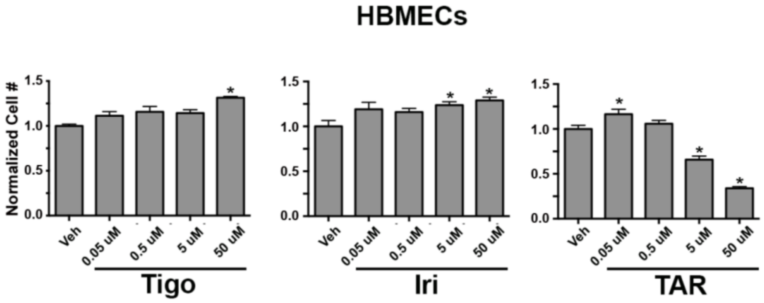

Supplemental Figure 4. Effect of Lead Compounds in HBMEC monocultures: HBMECs in monoculture were treated with Iridin, Tigogenin, or TAR for 4 days. Shown are cell numbers normalized to vehicle treated controls. * indicates p<0.05 vs. vehicle using one-way ANOVA analysis with Tukey's multiple comparisions test. Shown are mean and SEM.

Supplemental Table 1: Agents whose cytotoxicity was blocked by co-culture

Compound	Possible molecular mechanism(s)
2,6-DIMETHOXYQUINONE	DNA damaging agent
METHYLBENZETHONIUM CHLORIDE	Increases cytosolic calcium/apoptosis
CHLOROACETOXYQUINOLINE	Toxic to neural stem cells
CHLOROXINE	Inhibits ROR γ transcriptional activity
MENADIONE	Toxic to neural stem cells
SULCONAZOLE	DNA damaging agent
OXYPHENBUTAZONE	IFNα/β receptor antagonist
DIHYDROCELASTROL	Induces apoptosis/inhibits proteasomes
PERHEXILINE	Toxic to neural stem cells
BENZALKONIUM CHLORIDE	DNA damaging agent
TOTAROL	Inhibits Chain A, Human Bcl2-A1
HYDROQUINONE	DNA damaging agent
DIGITONIN	Detergent, water-solubilizes lipids
